# Neurological diseases and COVID-19: prospective analyses using the UK Biobank

**DOI:** 10.1007/s13760-021-01693-3

**Published:** 2021-05-05

**Authors:** Nicola Veronese, Lee Smith, Mario Barbagallo, Gianluigi Giannelli, Maria Gabriella Caruso, Anna Maria Cisternino, Maria Notarnicola, Chao Cao, Thomas Waldhoer, Lin Yang

**Affiliations:** 1 Geriatric Unit, Department of Internal Medicine and Geriatrics, University of Palermo, Palermo, Italy; 2 The Cambridge Centre for Sport and Exercise Sciences, Anglia Ruskin University, Cambridge, UK; 3 National Institute of Gastroenterology “S. de Bellis”, Research Hospital, Castellana Grotte, Italy; 4 Program in Physical Therapy, Washington University School of Medicine, St. Louis, MO USA; 5 Center for Human Nutrition, Washington University School of Medicine, St. Louis, MO USA; 6 Department of Epidemiology, Centre for Public Health, Medical University of Vienna, Vienna, Austria; 7 Department of Cancer Epidemiology and Prevention Research, Cancer Control Alberta, Alberta Health Services, Calgary, Canada; 8 Departments of Oncology and Community Health Sciences, University of Calgary, Calgary, Canada

**Keywords:** COVID-19, Neurological conditions, UK Biobank

## Abstract

COVID-19 (Coronavirus disease-19) may present with neurological signs, but whether people already affected by neurological conditions are at a higher risk of contracting COVID-19 is still not known. We, therefore, aimed to investigate the association of previously diagnosed neurological conditions with COVID-19. 502,536 community-dwelling UK Biobank participants (54.4% male, mean age 56.6 ± 10.3 years) were included. Among these, 57,463 participants had a diagnosis of neurological conditions (11.43%) and a total of 1326 COVID-19-positive cases were identified (0.26%). Neurological conditions were identified through medical history and linkage to data on hospital admissions (ICD-10 code G00–G99). COVID-19 presence was diagnosed using the data provided by Public Health England. The association of previous diagnosis of neurological conditions with COVID-19 was evaluated through logistic regressions, adjusted for potential confounders, reported as odds ratios (ORs) with their 95% confidence intervals (CIs). Nerve, nerve root and plexus disorders (G50–G59) were the most common conditions identified. The presence of COVID-19 was almost doubled in neurological conditions compared to the general population (0.45 vs. 0.24%, *p* < 0.0001). Previously diagnosed neurological conditions were associated with 60% higher odds of COVID-19 positive in the multivariable-adjusted model (OR = 1.6, 95% CI 1.4–1.8). Other degenerative diseases of the nervous system, extrapyramidal and movement disorders, polyneuropathies and other disorders of the peripheral nervous system, cerebral palsy and other paralytic syndromes were significantly associated with a higher odds of COVID-19. The presence of neurological conditions was associated with a significantly higher likelihood of COVID-19 compared to the general population.

**Supplementary Information:**

The online version contains supplementary material available at 10.1007/s13760-021-01693-3.

## Introduction

Coronaviruses are common ribonucleic acid viruses. In human beings, these viruses may infect several systems, including respiratory and gastrointestinal systems [1]. Infection by several coronaviruses usually lead to mild, self-limiting upper respiratory tract infections [2]. However, other coronaviruses are associated with severe acute respiratory syndrome (SARS-CoV) and Middle East respiratory syndrome (MERS-CoV).

Neurological manifestations are also being reported, even if they should be considered as atypical presentation of this infectious disease [3]. The most common neurological manifestations include headache and dizziness, followed by delirium, that often is the first manifestation of COVID-19 in older adults [3, 4]. However, similar to other viruses, some literature has reported the presence of cerebrovascular events (such as stroke), Guillain–Barre syndrome, acute transverse myelitis, and acute encephalitis in people affected by COVID-19 [5]. Of importance, it is has been reported that neurological manifestations may precede the typical features of COVID-19 [6]. Finally, the vulnerability of some neurological conditions for severe forms of COVID-19 (and by inference the likelihood of symptomatic COVID-19) has already been demonstrated in various studies [3, 7, 8]. Therefore, all these studies suggest that the association between COVID-19 and neurological conditions could be significant.

At the same time, very little is known regarding the risk of COVID-19 in people already affected by neurological conditions. People having these medical conditions can be at a higher risk of COVID-19 since they are more frail [9] and they usually have more severe impairment in their immune system [10] than in the general population. Understanding whether people with neurological conditions are at higher risk of COVID-19 could be of importance to better recognise the presence of this novel infectious disease in neurological settings, and finally, protect these people from its possible sequelae.

Given this background, the present study aimed to investigate the risk of COVID-19 in people with previous diagnosis of neurological conditions compared to the general population in the UK Biobank, a large study involving more than 500,000 participants.

## Materials and methods

### Study population

This research is covered by generic ethical approval from the NHS Research Ethics Committee (Ref. 11/NW/0382) for UK Biobank research. The analyses presented here were approved within project 41,245 by the UK Biobank research committee on 03 May 2019. The data used for the aims of this paper were originally collected from 2007 [11]. Briefly, the UK Biobank is a large health-focused resource, which specifically aims to understand the importance of environmental factors, genetics and lifestyle impact upon a broad array of health outcomes. The recruitment phase was conducted through a formal mail invitation, sent to 9.2 million households. Of them, more than 500,000 individuals attended UK Biobank assessment centres, to provide informed consent and complete baseline assessments. This process included touchscreen questionnaires, in-person interviews, and physical health examinations (full details for the UK Biobank’s assessment processes are available elsewhere) [11].

### Exposure: neurological conditions

Participants in the UK Biobank living with neurological conditions were identified based on medical history and linkage to data on hospital admissions. Since June 2013, data on UK Biobank participants’ primary/main diagnoses were extracted from all their hospital inpatient records and coded according to the International Classification of Disease version-10 (ICD-10). In January 2019, data on participants’ secondary diagnoses were also extracted from their hospital inpatients records and coded according to the ICD-10. Both data fields were used to identify overall and specific neurological conditions as follows: G00–G09: inflammatory disease of the central nervous system; G10–G14: systemic atrophies primarily affecting the central nervous system; G20–G26: extrapyramidal and movement disorders; G30–G32: other degenerative diseases of the nervous system; G35–G37: demyelinating diseases of the central nervous system; G40–G47: episodic and paroxysmal disorders; G50–G59: nerve, nerve root and plexus disorders; G60–G65: polyneuropathies and other disorders of the peripheral nervous system; G70–G73: diseases of myoneural junction and muscle; G80–G83: cerebral palsy and other paralytic syndromes; G89–G99: other disorders of the nervous system. Details of the algorithms used to combine the data from different sources to identify primary or secondary diagnosis have been described previously and are freely available on the UK Biobank website (www.ukbiobank.ac.uk).

### COVID-19 diagnosis

Baseline data from UK Biobank were linked to COVID-19 test results provided by Public Health England [12, 13], including the specimen date, origin (whether the person was an inpatient or not) and result (positive or negative). Confirmed COVID-19 was defined as at least one positive test result. Data were available for the period 16th March 2020 to 25th May 2020. The specimen were collected from an acute (emergency) care provider, an A&E department, an inpatient location, or resulted from health care associated infection, therefore, including symptomatic patients requiring hospital admission, or general inpatient screening, which includes asymptomatic patients.

### Covariates

A range of sociodemographic, lifestyle behaviour factors and medical conditions were included as covariates. Data were extracted on age (continuous), sex (male, female), BMI (normal, overweight, obese, missing), household income (less than £18,000, £18,000 to £30,999, £31,000 to £51,999, £52,000 to £100,000, greater than £100,000, missing), country of birth (UK, oversea, missing), and ethnicity (White, mixed, Asian or Asian British, Black or Black British, Chinese, others, missing). Information on age, sex, income, and country of birth were collected using a computerised questionnaire at UK Biobank assessment centres. BMI was calculated by body weight (kg)/height (metre)^2^, both of which were measured by a research assistant during physical health assessments.

In agreement with the American Heart Association (AHA) guidelines [14], the present study calculated a dietary score to assess healthy dietary behaviour. Briefly, for constructing this score, the UK Biobank participants completed a questionnaire on their habitual dietary intake. To determine a categorical healthy diet variable for the purposes of our analysis, the UK Biobank’s dietary intake data on the consumption of fruit, vegetables, fish and processed and red meats were extracted. A healthy diet was defined as adherence to at least two of the healthy food items that include total fruit and vegetable intake, total fish intake, and a low intake of processed and red meat [14]. Physical activity was assessed with the International Physical Activity Questionnaires (IPAQ) short form [15]. Based on the IPAQ scoring system, physical activity levels were defined as low, moderate, and high in the UK Biobank. Smoking status was defined in the UK Biobank as non-, past, and current smokers; likewise, alcohol intake was defined as non-, past, and current drinkers. In addition, previous diagnosis of medical conditions were considered as presence of diabetes, cancer, fractures, or other (other than diabetes, cancer, fracture) serious medical condition/disability.

### Statistical analysis

Participant characteristics including neurological condition were summarised using means and standard deviation for age, and frequencies and percentages for other variables. The distributions of participant characteristics were compared by neurological condition status using linear regression for continuous variables and Chi square tests for categorical variables. Sample size and prevalence of neurological conditions in the UK Biobank were presented for all and each specific neurological condition. In addition, proportions of specific neurological conditions among overall neurological conditions were presented.

Univariate, age-adjusted and multivariable-adjusted logistic regressions were carried out to estimate associations between existing neurological conditions and positive COVID-19 test results. Multivariable models adjusted for age, sex, BMI, household income, country of birth, dietary score, physical activity, smoking and alcohol drinking behaviour, and other medical conditions. Covariates with missing data were included in the model as missing categories. Furthermore, specific associations of eleven neurological conditions with positive COVID-19 test results were estimated using univariate, age-adjusted and multivariable-adjusted logistic regression models. In each neurological condition specific model, participants with the other ten neurological conditions were removed from the analysed sample to avoid biased estimations. All statistical analyses were conducted in Stata 14.0 (StataCorp, Texas, USA).

## Results

In total, 502,536 UK Biobank participants (54.4% male, mean age 56.6 ± 10.3 years) were included in this analysis. Among these, 57,463 participants had a diagnosis of neurological conditions (11.43%), and a total of 1326 COVID-19-positive cases were identified (0.26%).

As shown in Table 1, participants with neurological conditions, compared with those without prior neurological conditions, were more likely to be obese (34.9% vs. 23.0%), had household income less than £18,000 (27.3% vs. 18.3%), had low level of physical activity (18.4% vs. 14.7%), currently smoke (13.1% vs. 10.2%) and had other medical conditions (53.8% vs. 32.4%). The adherence to a healthy dietary score was significantly higher in people without neurological conditions. The proportion of participants who tested positive for COVID-19 were 0.24% (*n* = 1066) and 0.45% (*n* = 260) in those without and with neurological conditions, respectively (*p* < 0.0001 for all these comparisons).

**Table 1:** Sample characteristics of participants diagnosed with neurological conditions in the UK Biobank study (data reported in percentages, unless otherwise noted)

	Total (*n* = 502,536)	Neurological condition diagnoses		*P* values
		No (*n* = 445,073)	Yes (*n* = 57,463)	
Age, years^a^	56.6 (10.3)	56.4 (10.3)	58.1 (10.3)	< 0.001
Sex				0.888
Male	54.4	54.4	54.3	
Female	45.6	45.5	45.7	
Body mass index				< 0.001
Normal	32.8	33.9	24.4	
Overweight	42.2	42.6	39.1	
Obese	24.3	23.0	34.9	
Missing	0.6	0.5	1.6	
Household income				< 0.001
Less than £18,000	19.3	18.3	27.3	
£18,000–£30,999	21.5	21.4	22.2	
£31,000–£51,999	22.0	22.5	18.5	
£52,000–£100,000	17.2	17.9	11.2	
Greater than £100,000	4.6	4.9	2.3	
Missing	15.4	15.0	18.5	
Country of birth				< 0.001
UK	91.7	91.6	92.5	
Oversea	7.9	8.1	7.1	
Missing	0.4	0.3	0.4	
Ethnicity				< 0.001
White	94.1	94.0	94.2	
Mixed	0.6	0.6	0.6	
Asian or Asian British	4.6	1.6	1.5	
Black or Black British	2.0	2.0	1.9	
Chinese	0.3	0.3	0.2	
Others	0.9	0.9	0.9	
Missing	0.5	0.6	0.7	
Dietary score^b^				< 0.001
0	7.5	7.4	8.2	
1	31.7	31.7	31.9	
2	42.4	42.6	41.2	
3	18.2	18.2	18.5	
Missing	0.2	0.2	0.2	
Physical activity (IPAQ)				< 0.001
Low	15.2	14.7	18.5	
Moderate	32.6	33.1	29.0	
High	32.3	32.7	28.9	
Missing	19.9	19.5	23.6	
Smoking behaviour				< 0.001
Non-smoker	54.4	55.2	48.2	
Past smoker	34.4	34.0	37.8	
Current smoker	10.5	10.2	13.1	
Missing	0.6	0.6	0.9	
Alcohol drinking behaviour				< 0.001
Non-drinker	4.5	4.3	6.0	
Past drinker	3.6	3.2	6.5	
Current drinker	91.6	92.2	87.0	
Missing	0.3	0.3	0.5	
Medical condition^c^				< 0.001
No	65.2	67.6	46.2	
Yes	34.8	32.4	53.8	
COVID positive				< 0.001
Yes	0.26	0.24	0.45	

^a^Reported data: mean (standard deviation)

^b^Dietary score was derived based on the American Heart Association guideline, range 0–4, with higher score indicates healthier diet

^c^Including diabetes, cancer, fracture, and any other serious medical conditions/disability

Table 2 shows the sample sizes for the overall and each specific neurological condition as primary or secondary conditions in the UK Biobank study population prior to the COVID pandemic. The most common neurological condition was nerve, never root and plexus disorders (*n* = 24,984, 43.5% of all neurological conditions, and 4.97% of the UK Biobank study population), followed by episodic and paroxysmal disorder (*n* = 20,915, 36.4% of all neurological conditions, and 4.16% of the UK Biobank study population), which comprised over 70% of all neurological conditions. Other specific neurological conditions were generally around or below 5% of all diagnoses.

**Table 2:** Sample size and prevalence of specific neurological conditions in the UK biobank (total *n* = 502,536)

	*n*^a^	% In neurological conditions^a^	% In overall population^a^
Overall	57,463	100	11.43
Specific neurological condition			
Inflammatory diseases of the central nervous system (G00–G09)	918	1.6	0.18
Systemic atrophies primarily affecting the central nervous system (G10–G14)	643	1.1	0.13
Extrapyramidal and movement disorders (G20–G26)	3340	5.81	0.66
Other degenerative diseases of the nervous system (G30–G32)	1969	3.43	0.39
Demyelinating diseases of the central nervous system (G35–G37)	2032	3.54	0.40
Episodic and paroxysmal disorders (G40–G47)	20,915	36.4	4.16
Nerve, nerve root and plexus disorders (G50–G59)	24,984	43.5	4.97
Polyneuropathies and other disorders of the peripheral nervous system (G60–G65)	3027	5.27	0.60
Diseases of myoneural junction and muscle (G70–G73)	766	1.33	0.15
Cerebral palsy and other paralytic syndromes (G80–G83)	3491	6.08	0.69
Other disorders of the nervous system (G89–G99)	6317	10.99	1.26

^a^Proportions of specific neurological conditions add up higher than the overall due to neurological conditions

Table 3 presents the associations of neurological condition diagnoses with COVID-19-positive results in the UK Biobank. Living with neurological conditions was associated with nearly a twofold higher odds of COVID-19 positivity in the univariate (OR = 1.9, 95% CI 1.7–2.2) and age-adjusted (OR = 1.9, 95% CI 1.7–2.2) models, which remained significant in the multivariable-adjusted model (OR = 1.6, 95% CI 1.4–1.8) after adjusting for age, sex, BMI, household income, country of birth, dietary score, physical activity smoking and alcohol drinking behaviour and other medical conditions (for full results of logistic regression, see Supplementary Table 1).

**Table 3:** Univariate, age-adjusted and multivariable-adjusted associations of neurological conditions with COVID-19-positive diagnosis in the UK Biobank (total *n* = 502,536)

	Univariate			Age-adjusted^a^			Multivariable-adjusted^b^		
Overall	1.9	(1.7–2.2)	< 0.001	1.9	(1.7–2.2)	< 0.001	1.6	(1.4–1.8)	< 0.001
Specific neurological condition^c^									
Inflammatory diseases of the central nervous system (G00–G09)	2.3	(0.9–5.5)	0.067	2.3	(1.0–5.6)	0.064	2.0	(0.8–4.7)	0.136
Systemic atrophies primarily affecting the central nervous system (G10–G14)	2.0	(0.6–6.1)	0.248	2.0	(0.6–6.3)	0.228	1.8	(0.6–5.6)	0.315
Extrapyramidal and movement disorders (G20–G26)	3.5	(2.4–5.1)	< 0.001	3.7	(2.5–5.4)	< 0.001	3.2	(2.2–4.7)	< 0.001
Other degenerative diseases of the nervous system (G30–G32)	7.1	(5.0–10.1)	0.000	7.5	(5.3–10.7)	< 0.001	6.3	(4.4–9.0)	< 0.001
Demyelinating diseases of the central nervous system (G35–G37)	1.6	(0.8–3.3)	0.161	1.6	(0.8–3.3)	0.171	1.6	(0.8–3.2)	0.215
Episodic and paroxysmal disorders (G40–G47)	1.9	(1.5–2.3)	< 0.001	1.9	(1.5–2.3)	< 0.001	1.5	(1.2–1.9)	< 0.001
Nerve, nerve root and plexus disorders (G50–G59)	1.5	(1.2–1.9)	< 0.001	1.5	(1.2–1.9)	< 0.001	1.3	(1.1–1.6)	0.013
Polyneuropathies and other disorders of the peripheral nervous system (G60–G65)	3.7	(2.6–5.5)	< 0.001	3.9	(2.6–5.7)	< 0.001	2.9	(2.0–4.3)	< 0.001
Diseases of myoneural junction and muscle (G70–G73)	1.6	(0.5–5.1)	0.394	1.7	(0.5–5.2)	0.377	1.3	(0.4–4.2)	0.613
Cerebral palsy and other paralytic syndromes (G80–G83)	3.7	(2.6–5.3)	< 0.001	3.8	(2.7–5.5)	< .001	2.9	(2.0–4.2)	< 0.001
Other disorders of the nervous system (G89–G99)	1.5	(1.0–2.2)	0.082	1.5	(1.0–2.3)	0.071	1.2	(0.8–1.9)	0.323

^a^Models adjusted for age only

^b^Models adjusted for age, sex, BMI, household income, country of birth, ethnicity, dietary score (based on the American Heart Association recommendation), physical activity, smoking behaviour, alcohol drinking behaviour, and medical conditions including diabetes, cancer, fracture, and any other serious medical conditions/disability

^c^Models for each specific neurological condition excluded other neurological conditions

**Fig. 1:**
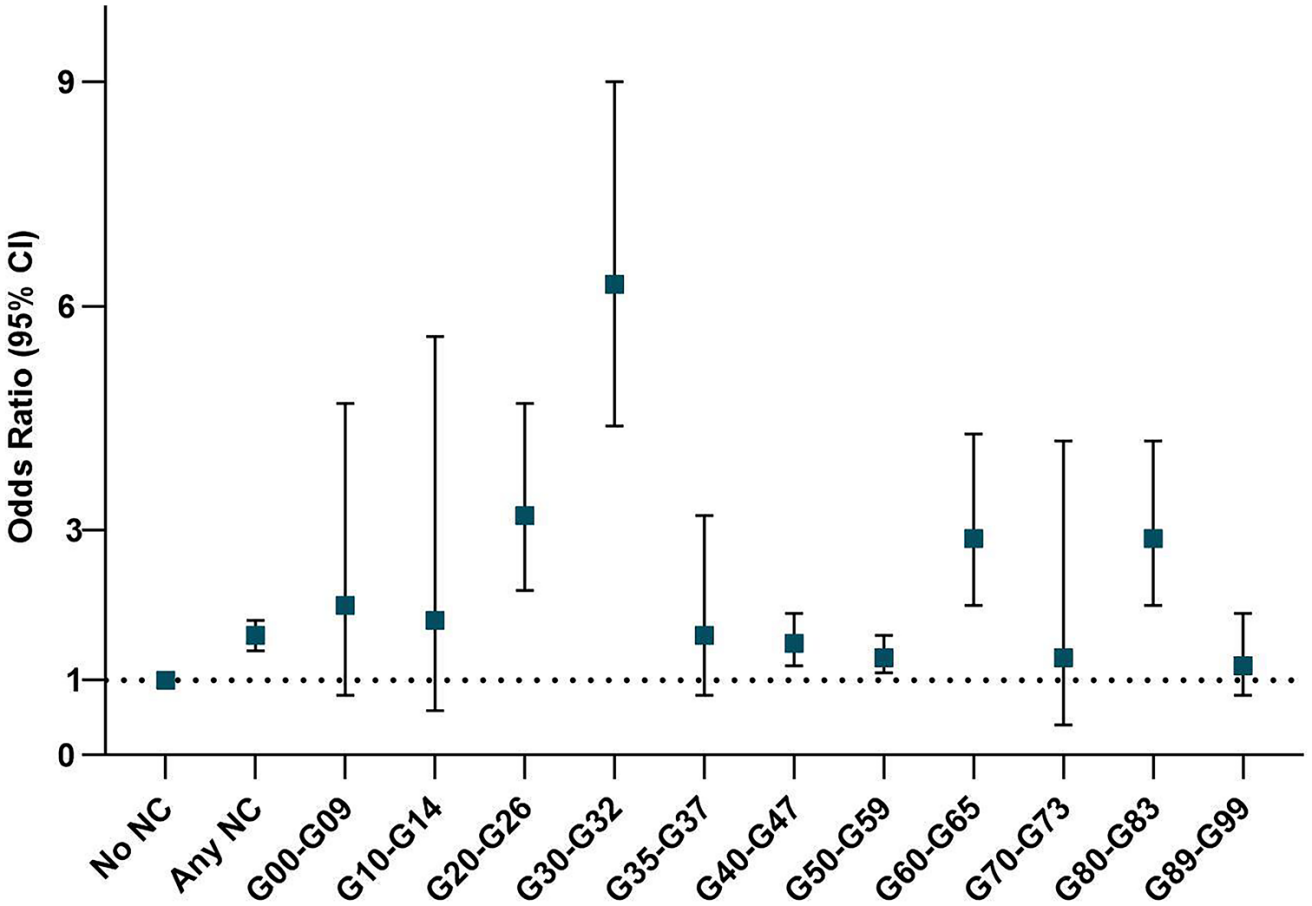
Multivariable-adjusted odds ratios of neurological conditions with COVID-19-positive diagnosis in the UK Biobank (total *n* = 502,536)^a,b^. *NC* Neurological condition, *G00–G09* Inflammatory diseases of the central nervous system, *G10–G14* Systemic atrophies primarily affecting the central nervous system, *G20–G26* Extrapyramidal and movement disorders, *G30–G32* Other degenerative diseases of the nervous system, *G35–G37* Demyelinating diseases of the central nervous system, *G40–G47* Episodic and paroxysmal disorders, *G50–G59* Nerve, nerve root and plexus disorders, *G60–G65* Polyneuropathies and other disorders of the peripheral nervous system, *G70–G73* Diseases of myoneural junction and muscle, *G80–G83* Cerebral palsy and other paralytic syndromes, *G89–G99* Other disorders of the nervous system. ^a^Odds ratios were adjusted for age, sex, BMI, household income, country of birth, dietary score (based on the American Heart Association recommendation), physical activity, smoking behaviour, alcohol drinking behaviour, and medical conditions including diabetes, cancer, fracture, and any other serious medical conditions/disability. ^b^Odds ratios for each specific neurological condition excluded other neurological conditions

To evaluate whether this association may vary between different types of neurological condition diagnoses, logistic regression models were carried out for each specific neurological condition (for specific OR and 95% CI, see Table 3 and Fig. 1). In the multivariable-adjusted models, other degenerative diseases of the nervous system (G30–G32) (multivariable-adjusted OR = 6.3, 95% CI 4.4 to 9.0) were associated with sixfold higher odds of positive COVID-19 test results. In addition, extrapyramidal and movement disorders (G20–G26) (multivariable-adjusted OR = 3.2, 95% CI 2.2–4.7), polyneuropathies and other disorders of the peripheral nervous system (G60–G65) (multivariable-adjusted OR = 2.9, 95% CI 2.0–4.3), and cerebral palsy and other paralytic syndromes (G80–G83) (multivariable-adjusted OR = 2.9, 95% CI 2.2–4.2) were associated with approximately threefold higher odds of positive COVID-19 test results. In multivariable-adjusted logistic regression models of aforementioned neurological conditions, *p* values remained significant after Bonferroni correction for multiple testing (data not shown). These associations were either non-significant or with ORs smaller than 2 for other specific neurological conditions.

## Discussion

In this large prospective study carried out using UK Biobank participants, living with previously diagnosed neurological conditions was associated with 60% higher odds of COVID-19-positive testing. The higher odds of COVID-19 were particularly significant in some neurological conditions, including extrapyramidal and movement disorders, polyneuropathies, and paralysis/cerebral palsy.

People with neurological diseases had a higher proportion of obesity, were poorer, but had higher proportion of people being physically active and a better attitude in healthier lifestyle, even if they were more frequently smokers than their counterparts, making them at higher risk of COVID-19 infection. To the best of our knowledge, this is the first study to report a prospective association of neurological conditions with COVID-19. The association between COVID-19 and neurological disorders appears strong, as suggested by the recent literature. For example, data from 214 patients with COVID-19 reported neurological symptoms in at least one third of the patients [6]. The data reported so far suggests that COVID-19 infected patients with more severe systemic presentations were more likely to have neurologic symptoms, compared with those with milder forms of the infection [6]. Neurological conditions, by themselves, have a great epidemiological impact [16] and they are associated with a high risk of disability [17] and poor quality of life [18]. At the same time, the infection from COVID-19 may further worsen the clinical course of these conditions and possibly lead to higher mortality risk.

In the present study, data suggests that some specific neurological conditions are more likely to be affected by COVID-19. In particular, compared to people free of neurological conditions, the odds of SARS-CoV-2 infection were three times higher, after considering potential confounders, in people affected by extrapyramidal and movement disorders, a category that included Parkinson’s disease. Some studies, contrary to our findings, reported that there is no evidence, or lack of evidence, suggesting that patients with Parkinson’s disease may have a similar risk of contracting COVID-19 to general population [19, 20]. However, the small sample sizes and the retrospective design of these studies limit these findings.

In addition, the present study found that COVID-19 was more prevalent in people affected by polyneuropathies and other disorders of the peripheral nervous system than in the general population. Again, the association between COVID-19 and these specific neurological conditions is intriguing. In an observational study of 214 patients with confirmed diagnoses of COVID-19, approximately 10% presented with peripheral nervous system symptoms [21]. These symptoms may include hypogeusia, hyposmia, hypoplasia and neuralgia that now are increasingly recognised as highly specific for COVID-19 [22]. Moreover, the category of polyneuropathies and other disorders of the peripheral nervous system included Guillain-Barré Syndrome which may be a consequence not only of COVID-19, but also of other viral infections [23]. Finally, the present study reported a significant higher odds of COVID-19 in participants affected by cerebral palsy and other paralytic syndromes comparing to those free of neurological conditions. To the best of our knowledge, this is the first report to highlight this association. In this already “at risk” population, COVID-19 may have significant detrimental consequences in comparison to the general population. Further research to confirm or refute the present findings and to further explain potential underlying mechanisms is warranted.

The present study has several strengths and limitations. The strengths of the UK Biobank include its extensive phenotyping which enables the adjustment for demographic and lifestyle risk factors, disease and ill health, and its large sample size [13]. However, it is not representative of the general population living in the UK. For example, it has been estimated that the overall prevalence of COVID-19 in the United Kingdom is 0.55% [24], whilst we found an overall presence of 0.26%. Nevertheless, the prospective nature of the UK Biobank study was able to document neurological condition diagnoses prior to the COVID-19 pandemic, facilitating certain levels of temporal association. Moreover, we did not include information regarding medications. For example, many neurological diseases require immunosuppressive medications that are obviously associated with more severe forms of COVID-19. Furthermore, the COVID-19 testing was not implemented in the overall population. However, the criteria of COVID-19 testing in the UK are primarily based on symptoms. Given this, we are unable to distinguish whether higher likelihood of SARS-CoV-2 infection being symptomatic versus asymptomatic in certain neurological conditions or a higher susceptibility to acquiring the infection from people affected by neurological diseases. Finally, the observed higher COVID-19 positivity in neurological diseases could be due to higher likelihood of SARS-CoV-2 infection being symptomatic versus asymptomatic in certain neurological conditions, such as extrapyramidal and movement disorders, polyneuropathies and paralysis/cerebral palsy. Alternatively, our observed association might have been due to a greater susceptibility of COVID-19 among people with neurological conditions because they may experience limited self-cleanliness and other aspects of grooming behaviour, poor adherence to social distancing or shielding and other aspects of social behaviour associated with nursing home residence, carer visits, and reliance on public transport.

In conclusion, this study shows that the people affected by neurological conditions have a significantly higher likelihood of COVID-19 in comparison to the general population. In particular, this investigation highlights that people with some common specific neurological conditions (such as neurological paralyses, polyneuropathies, and extrapyramidal disorders) may be at a greater risk of COVID-19. These findings need to be confirmed in future studies. Given the added neurological manifestations associated with COVID-19, the prognosis of neurological patients with COVID-19 warrants further investigation.

## Supplementary Information

Below is the link to the electronic supplementary material.

Supplementary file1 (DOCX 26 kb)
