# Supplementary material for: Neurological diseases and COVID-19: prospective analyses using the UK Biobank
Source: Acta Neurol Belg. 2021 May 5;121(5):1295–303. doi: 10.1007/s13760-021-01693-3 (PMC8098789; doi:10.1007/s13760-021-01693-3)
Supplement: Supplementary file 1 — Supplementary file1 (DOCX 26 kb) [file 13760_2021_1693_MOESM1_ESM.docx]

| **Supplementary table 1: Univariate, age-adjusted and multivariable-adjusted associations of neurological conditions with COVID-19 positive diagnosis in the UK Biobank (total n=502,536)** | | | | | | | | | | | | | | |
| --- | --- | --- | --- | --- | --- | --- | --- | --- | --- | --- | --- | --- | --- | --- |
|  | **Univariate** | | | |  | **Age-adjusted** | | | |  | **Multivariable-adjusted** | | | |
|  | OR (95% CI) | | | P-value |  | OR (95% CI) | | | P-value |  | OR (95% CI) | | | P-value |
| **Neurological conditions** |  |  |  |  |  |  |  |  |  |  |  |  |  |  |
| No | Reference | | |  |  | Reference | | |  |  | Reference | | |  |
| Yes | 1.9 | (1.7 to | 2.2) | <.001 |  | 1.9 | (1.7 to | 2.2) | <.001 |  | 1.6 | (1.4 to | 1.8) | 0.000 |
| **Age, years^1^** |  |  |  |  |  | 1.0 | (1.0 to | 1.0) | 0.425 |  | 1.0 | (1.0 to | 1.0) | 0.013 |
| **Sex** |  |  |  |  |  |  |  |  |  |  |  |  |  |  |
| Male |  |  |  |  |  |  |  |  |  |  | Reference | | |  |
| Female |  |  |  |  |  |  |  |  |  |  | 1.3 | (1.2 to | 1.5) | 0.000 |
| **Body mass index** |  |  |  |  |  |  |  |  |  |  |  |  |  |  |
| Normal |  |  |  |  |  |  |  |  |  |  | Reference | | |  |
| Overweight |  |  |  |  |  |  |  |  |  |  | 1.2 | (1.1 to | 1.4) | 0.003 |
| Obese |  |  |  |  |  |  |  |  |  |  | 1.6 | (1.4 to | 1.8) | 0.000 |
| Missing |  |  |  |  |  |  |  |  |  |  | 1.5 | (0.9 to | 2.6) | 0.129 |
| **Household income** |  |  |  |  |  |  |  |  |  |  |  | <.001 |  |  |
| Less than £18,000 |  |  |  |  |  |  |  |  |  |  | Reference | | |  |
| £18,000 to £30,999 |  |  |  |  |  |  |  |  |  |  | 0.7 | (0.6 to | 0.9) | 0.000 |
| £31,000 to £51,999 |  |  |  |  |  |  |  |  |  |  | 0.7 | (0.6 to | 0.8) | 0.000 |
| £52,000 to £100,000 |  |  |  |  |  |  |  |  |  |  | 0.6 | (0.5 to | 0.8) | 0.000 |
| Greater than £100,000 |  |  |  |  |  |  |  |  |  |  | 0.5 | (0.4 to | 0.7) | 0.000 |
| Missing |  |  |  |  |  |  |  |  |  |  | 0.9 | (0.7 to | 1.0) | 0.129 |
| **Country of birth** |  |  |  |  |  |  |  |  |  |  |  |  |  |  |
| UK |  |  |  |  |  |  |  |  |  |  | Reference | | |  |
| Oversea |  |  |  |  |  |  |  |  |  |  | 1.1 | (0.9 to | 1.4) | 0.311 |
| Missing |  |  |  |  |  |  |  |  |  |  | 0.9 | (0.4 to | 2.3) | 0.827 |
| **Ethnicity** |  |  |  |  |  |  |  |  |  |  |  |  |  |  |
| White |  |  |  |  |  |  |  |  |  |  | Reference | | |  |
| Mixed |  |  |  |  |  |  |  |  |  |  | 1.2 | (0.6 to | 2.3) | 0.639 |
| Asian or Asian British |  |  |  |  |  |  |  |  |  |  | 2.0 | (1.4 to | 2.9) | 0.000 |
| Black or Black British |  |  |  |  |  |  |  |  |  |  | 2.9 | (2.2 to | 3.9) | 0.000 |
| Chinese |  |  |  |  |  |  |  |  |  |  | 1.7 | (0.7 to | 3.9) | 0.225 |
| Others |  |  |  |  |  |  |  |  |  |  | 1.7 | (1.1 to | 2.6) | 0.030 |
| Missing |  |  |  |  |  |  |  |  |  |  | 1.4 | (0.7 to | 2.9) | 0.357 |
| **Dietary score** |  |  |  |  |  |  |  |  |  |  |  |  |  |  |
| 0 |  |  |  |  |  |  |  |  |  |  | 1.0 | (0.8 to | 1.2) | 0.647 |
| 1 |  |  |  |  |  |  |  |  |  |  | 0.9 | (0.8 to | 1.1) | 0.472 |
| 2 |  |  |  |  |  |  |  |  |  |  | 1.0 | (0.8 to | 1.2) | 0.873 |
| 3 |  |  |  |  |  |  |  |  |  |  | 0.5 | (0.1 to | 3.0) | 0.454 |
| Missing |  |  |  |  |  |  |  |  |  |  | 0.2 |  |  |  |
| **Physical activity (IPAQ)** |  |  |  |  |  |  |  |  |  |  |  |  |  |  |
| Low |  |  |  |  |  |  |  |  |  |  | Reference | | |  |
| Moderate |  |  |  |  |  |  |  |  |  |  | 0.9 | (0.8 to | 1.1) | 0.229 |
| High |  |  |  |  |  |  |  |  |  |  | 1.0 | (0.8 to | 1.2) | 0.963 |
| Missing |  |  |  |  |  |  |  |  |  |  | 1.2 | (1.0 to | 1.4) | 0.073 |
| **Smoking behaviour** |  |  |  |  |  |  |  |  |  |  |  |  |  |  |
| Non-smoker |  |  |  |  |  |  |  |  |  |  | Reference | | |  |
| Past smoker |  |  |  |  |  |  |  |  |  |  | 1.3 | (1.1 to | 1.5) | 0.000 |
| Current smoker |  |  |  |  |  |  |  |  |  |  | 1.0 | (0.9 to | 1.2) | 0.786 |
| Missing |  |  |  |  |  |  |  |  |  |  | 1.4 | (0.8 to | 2.8) | 0.259 |
| **Alcohol drinking behaviour** |  |  |  |  |  |  |  |  |  |  |  |  |  |  |
| Non-drinker |  |  |  |  |  |  |  |  |  |  | Reference | | |  |
| Past drinker |  |  |  |  |  |  |  |  |  |  | 1.1 | (0.8 to | 1.5) | 0.568 |
| Current drinker |  |  |  |  |  |  |  |  |  |  | 0.8 | (0.7 to | 1.0) | 0.104 |
| Missing |  |  |  |  |  |  |  |  |  |  | 0.9 | (0.3 to | 2.6) | 0.895 |
| **Medical condition** |  |  |  |  |  |  |  |  |  |  |  | <.001 |  |  |
| No |  |  |  |  |  |  |  |  |  |  | Reference | | |  |
| Yes |  |  |  |  |  |  |  |  |  |  | 1.2 | (1.1 to | 1.4) | 0.001 |
